# Supplementary figures and images for: Neutrophil-to-lymphocyte ratio as a prognostic indicator in COVID-19: Evidence from a northern tanzanian cohort
Source: PLoS One. 2025 Jan 31;20(1):e0300231. doi: 10.1371/journal.pone.0300231 (PMC11785306; doi:10.1371/journal.pone.0300231)

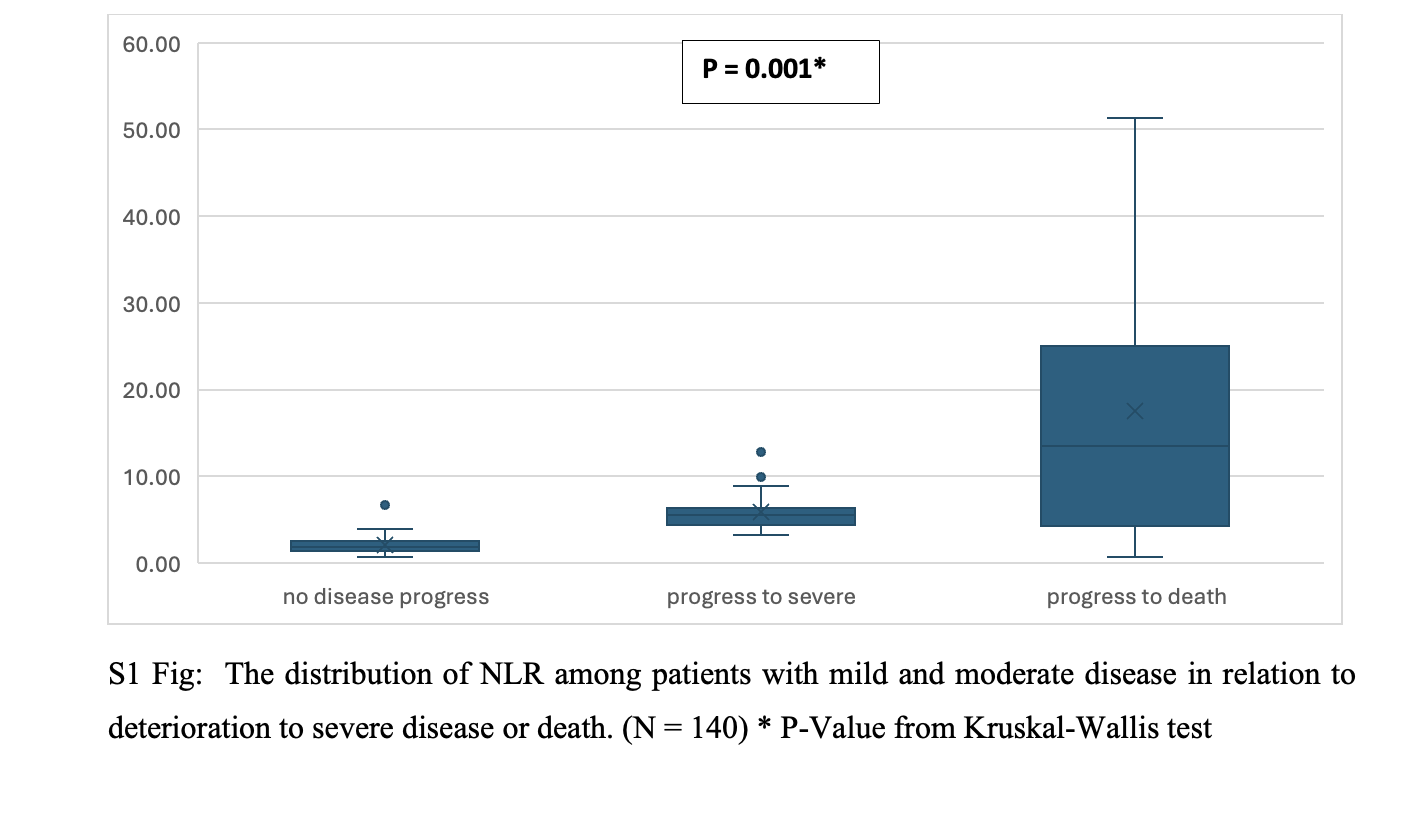

Supplement: S1 Fig — (TIF) [file pone.0300231.s001.tif]

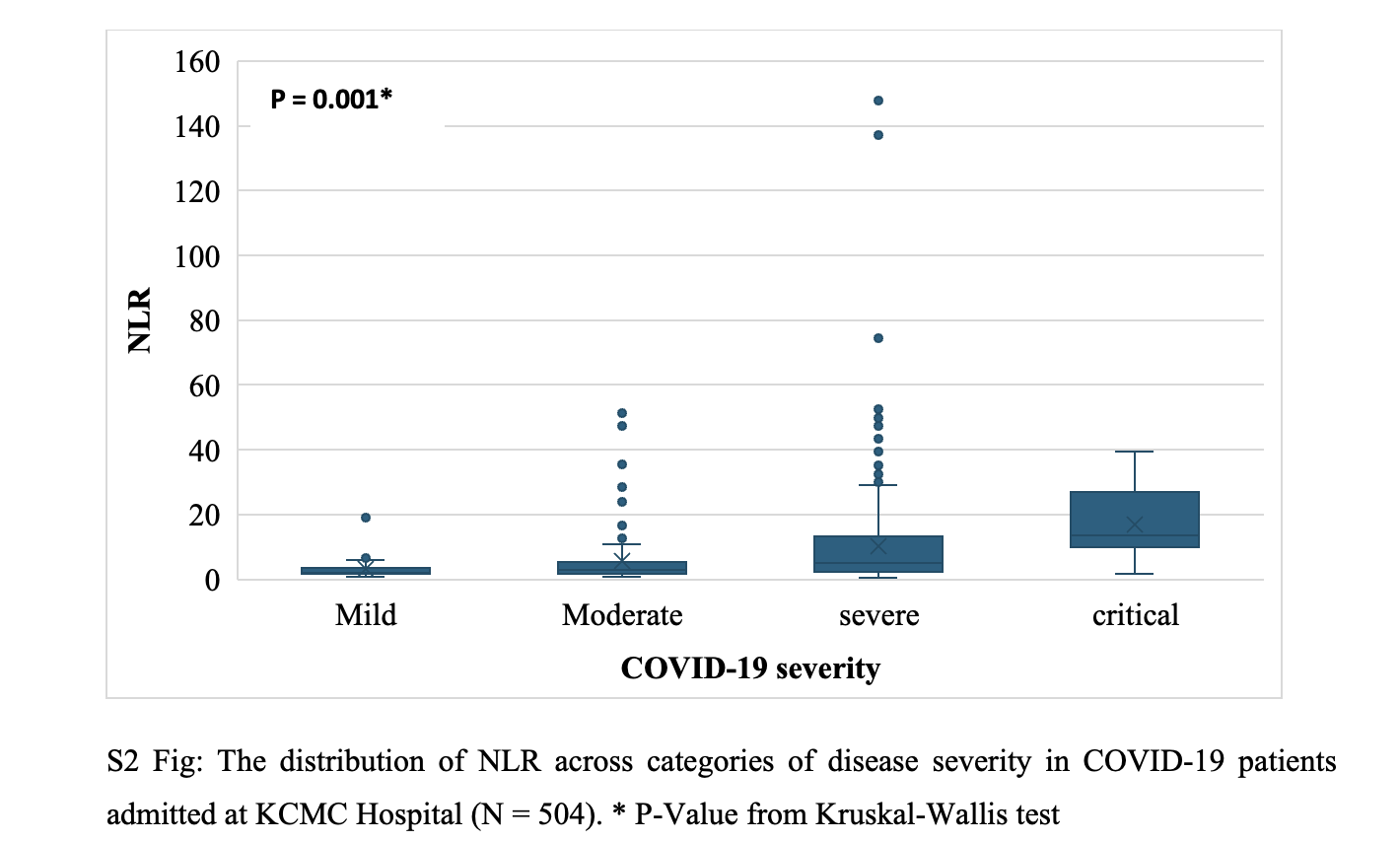

Supplement: S2 Fig — (TIF) [file pone.0300231.s002.tif]

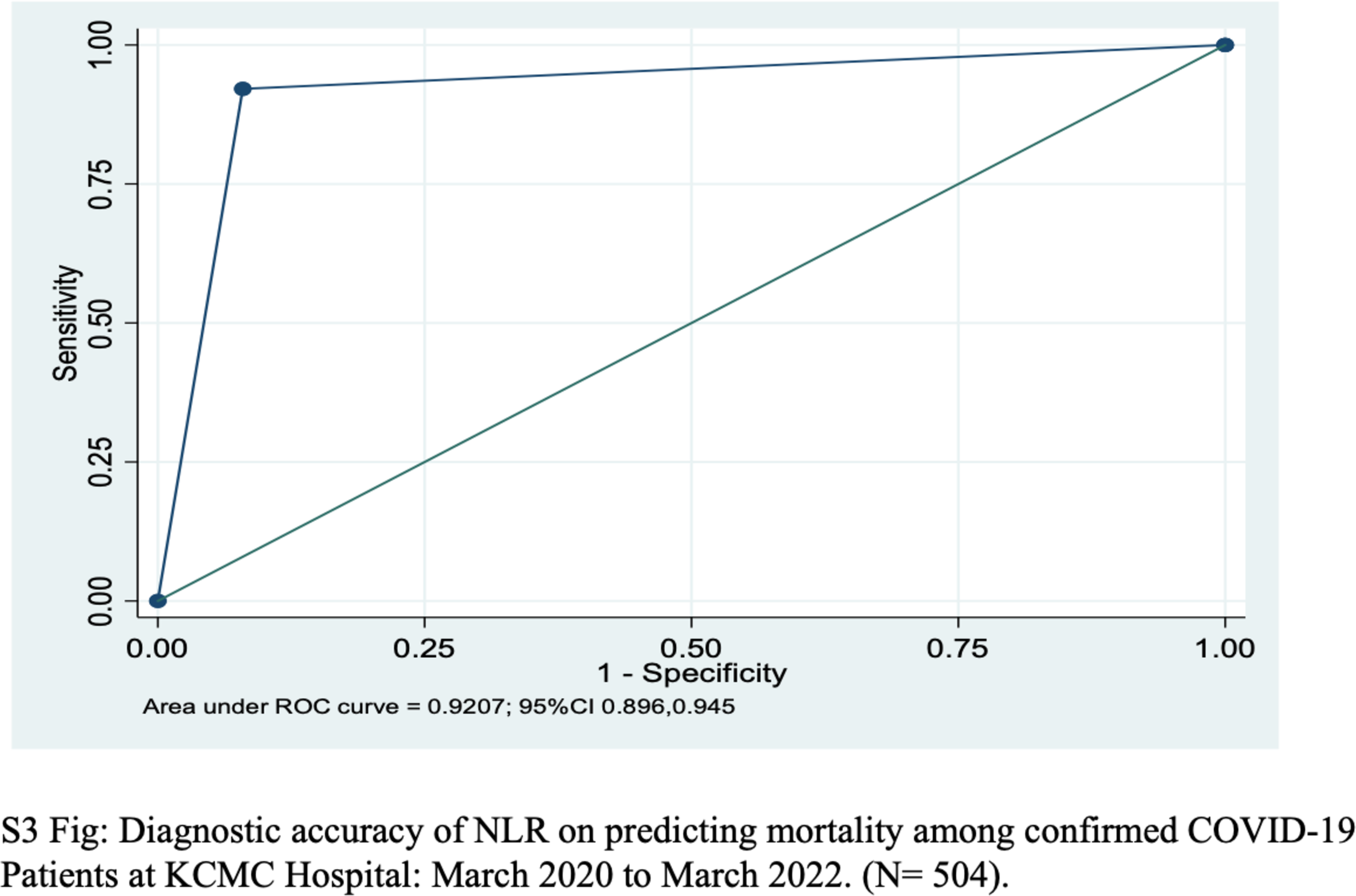

Supplement: S3 Fig — (TIF) [file pone.0300231.s003.tif]
