# Supplementary material for: Neutrophil-to-lymphocyte ratio as a prognostic indicator in COVID-19: Evidence from a northern tanzanian cohort
Source: PLoS One. 2025 Jan 31;20(1):e0300231. doi: 10.1371/journal.pone.0300231 (PMC11785306; doi:10.1371/journal.pone.0300231)
Supplement: Attachment — Submitted filename: Response to Reviewers .docx [file pone.0300231.s005.docx]

Norman Jonas Kyala,

P.O Box 3010,

Moshi.

normanjonasmd@gmail.com

26^th^ September 2024.

PLOS ONE Journal office, U.S Headquarters.

1265 Battery Street, suite 200,

San Francisco, CA 94111,

United States,

Dear Plosone journal reviewer(s)

REF: **RE-SUBMISSION FOR MANUSCRIPT PUBLICATION.**

Kindly refer to the heading above.

I kindly ask your attention to the revised manuscript ***titled "Neutrophil-to-Lymphocyte Ratio as a Prognostic Indicator in COVID-19: Evidence from a Northern Tanzanian Cohort"*** for potential inclusion in your esteemed journal.

As an Internal Medicine Specialist from Tanzania, I believe the findings presented in this manuscript will contribute significantly to our understanding of COVID-19 prognostic indicators in diverse clinical settings. I have taken great care in addressing the comments and suggestions provided by the academic editor and reviewers, to enhance the manuscript. The revisions have strengthened the arguments and clarified the implications of the research, aligning it with the high standards of your journal.

I appreciate your consideration of my manuscript and look forward to the opportunity to contribute to the scientific community through your publication. It’s my hope my request will be accepted and given high priority.

I hope my submission will be met with consideration and given the attention it deserves.

Yours Sincerely.

Norman Jonas Kyala

**Summary of Changes per Journal Requirements and reviewers’ comments**

| **Journal Requirements:** | |
| --- | --- |
| **Comment** | **Remark** |
| 1. When submitting your revision, we need you to address these additional requirements.  Please ensure that your manuscript meets PLOS ONE's style requirements, including those for file naming. | The comment has been addressed in the revised manuscript.  We ensured that our manuscript meets PLOS ONE's style requirements. |
| 2. Please provide additional details regarding participant consent. In the ethics statement in the Methods and online submission information, please ensure that you have specified (1) whether consent was informed and (2) what type you obtained (for instance, written or verbal, and if verbal, how it was documented and witnessed). If your study | The comment has been addressed on revised manuscript on page 8 line 201 to line 205  In addition, all data accessed for this study were fully anonymized before analysis to ensure participant confidentiality.  Kilimanjaro Christian Medical University  College Research and Ethics Review  Committee reviewed and approved our |
| included minors, state whether you obtained consent from parents or guardians. If the need for consent was waived by the ethics committee, please include this information.  If you are reporting a retrospective study of medical records or archived samples, please ensure that you have discussed whether all data were fully anonymized before you accessed them and/or whether the IRB or ethics committee waived the requirement for informed consent. If patients provided informed written consent to have data from their medical records used in research, please include this information. | protocol, including the anonymization process. |
| 3. In the online submission form, you indicated that [Data cannot be shared publicly because of government guide. Data are available from the KCMC Institutional Data Access / Ethics  Committee (contact via normanjonasmd@gmail.com) for researchers who meet the criteria for access to confidential data.]. All PLOS journals now require all data underlying the findings described in their manuscript to be freely available to other researchers, either 1. In a public repository, 2. Within the manuscript itself, | The comment has been addressed on online submission form and data has been shared as supplementary information. |
| 1.Please amend your list of authors on the manuscript to ensure that each author is linked to an affiliation. We note that you have included affiliation numbers 1,2,3, 4 and 5 however only affiliations 1,2, 3 and 4 have authors linked to them.  Please amend affiliation 5 to link an author to it or remove if added in error. | Thank you for this comment, affiliation 5 has been linked to an author – Innocent Mboya who is current full affiliated with Lund University - Sweden. |
| 2.Please include a copy of Table 3 which you refer to in your text on page 10. | Thank you for this observation; apologies that there was an error in naming the table 3 and table 4. In revised manuscript Table 3 is now found in Page 19 line 470 and table 4 is found in page 20 line 479. |
| or 3. Uploaded as supplementary information.  This policy applies to all data except where public deposition would breach compliance with the protocol approved by your research ethics board. If your data cannot be made publicly available for ethical or legal reasons (e.g., public availability would compromise patient privacy), please explain your reasons on resubmission and your exemption request will be escalated for approval. |  |
| 5. Please review your reference list to ensure that it is complete and correct. If you have cited papers that have been retracted, please include the rationale for doing so in the manuscript text, or remove these references and replace them with relevant current references. Any changes to the reference list should be mentioned in the rebuttal letter that accompanies your revised manuscript. If you need to cite a retracted article, indicate the article’s retracted status in the References list and also include a citation and full reference for the retraction notice. | The comment has been addressed on revised manuscript. |
| 3.Thank you for providing the following supporting information file: S1 Appendix - data.xlsxf  We note this file contains potentially identifying information such as specific ages, dates, and patient codes. Please remove all potentially identifying information and uploaded a completely anonymized data set. | Thank you for this comment, The comment has been addressed on revised on Data file |
| 4. Article is a good attempt. however other causes of NLR ratio derangement could have been elaborated better too. Please revise the article again. | Thank you for your feedback. We appreciate the opportunity to revise our manuscript. We understand the need for a more comprehensive discussion of factors that could affect the NLR ratio. Although our data does not include direct evidence of other causes of NLR derangement, we have expanded the discussion to acknowledge other commonly recognized factors and the rationale behind our exclusion criteria. This has been addressed in revised manuscript line 307 -317. |
|  |  |
| We've checked your submission and before we can proceed, we need you to address the following issues:  1. Please ensure that you refer to Table 3 in your text as, if accepted, production will need this reference to link the reader to the Table. | The comment has been addressed on revised manuscript. |

**Response to Reviewer 1**

| **Comment** | **Remark** |
| --- | --- |
| Methodology  1. l recommends providing further clarification on the utilization of ROC analysis for calculating sensitivity and specificity. | Thank you for your insightful comment. We appreciate the opportunity to provide further clarification on the utilization of ROC analysis in our study. The comment has been accomodated in revised manuscript page 7 line 176 to line 184.  For further clarification; ROC analysis is a widely used statistical method for evaluating the diagnostic performance of a biomarker or test. In our study, we employed ROC analysis to assess the diagnostic accuracy of the Neutrophil-to-Lymphocyte Ratio (NLR) in predicting mortality among COVID-19 patients. Several studies have also utilized ROC analysis to evaluate the prognostic value of NLR COVID-19 at various clinical settings including studies from Liu et al.  (2020), Qin et al. (2020) and Fois et al.  (2020).  In our study, we used the following approach:  1. We plotted the ROC curve by calculating the true positive rate (sensitivity) and the false positive rate (1 - specificity) at various threshold levels of NLR. Sensitivity measures the proportion of actual positives  (patients who died) correctly |

|  | identified by the NLR threshold, while specificity measures the proportion of actual negatives (patients who survived) correctly identified.   2. We calculated the Area Under the Curve (AUC). The AUC is a single scalar value that summarizes the overall diagnostic accuracy of the NLR. An AUC of 0.5 indicates no diagnostic ability, while an AUC of 1.0 indicates perfect diagnostic ability. In our study, the AUC was   0.9207 (95% CI 0.896–0.945).   3. We identified the optimal NLR threshold for predicting mortality by maximizing the Youden Index   (sensitivity + specificity - 1). The NLR cut-off in predicting mortality due to COVID-19 determined by ROC analysis was 6.10 with a sensitivity of 92.1% (95% CI 89.2%– 94.0%) and a specificity of 92.0% (95% CI 89.7%–94.4%). The high sensitivity and specificity values suggest that the NLR cut-off of 6.1 can be considered as a threshold for predicting the mortality of COVID-19 patients at admission.   4. The sensitivity and specificity values at the optimal threshold were reported |
| --- | --- |

|  | to indicate how well the NLR can predict mortality among COVID-19 patients during admission.  By utilizing ROC analysis, we ensured a statistically sound method for evaluating the diagnostic accuracy of NLR in our cohort. This methodology is well-established in the literature and provides a reliable means of determining the prognostic utility of biomarkers in clinical research.  We hope this detailed explanation clarifies our use of ROC analysis and reassures the validity of our findings. |
| --- | --- |
| 1. Regarding the first paragraph, it would be beneficial to compare the mortality rate and severity of illness in COVID-19 patients between the Romanian study and the current study. Is the threshold for the Romanian study one and a half times that of the current study? Please interpret the reasons for such discrepancies and highlight any methodological differences between the two studies.? | Thank you for this feedback; this comment has been addressed in the revised manuscript page 11 line 285 to line 292. |
| 2. In the second paragraph, instead of repeating individual studies, summarizing and referencing similar studies on the positive correlation between Higher NLR | Thank you for this feedback; this comment has been addressed in the revised manuscript page 11 on line 279 to line 282. |

| and severe outcomes in COVID-19 patients would suffice. |  |
| --- | --- |
| 2. Do you recommend healthcare professionals to assess NLR levels in COVID-19 positive patients during their visits? | Thank you for your important question.  Based on our findings, we do recommend that healthcare professionals assess NLR levels in COVID-19 positive patients during their admission in Northern Tanzania.  Our study demonstrated a significant prognostic value of NLR in predicting mortality among these patients. The ROC analysis revealed an NLR cut-off of 6.10, which was associated with a high sensitivity of 92.1% (95% CI 89.2%–94.0%) and specificity of 92.0% (95% CI 89.7%–94.4%). The AUC was 0.9207 (95% CI 0.896–0.945), indicating excellent diagnostic accuracy.  These results suggest that an NLR threshold of 6.1 can be used to predict mortality in COVID-19 patients at admission in Tanzania.  However multicentre, large prospective study is needed for validation in other settings. |
| 3. Your study focused only on hospitalized individuals, while outpatient cases were not considered. | Thank you for your valuable feedback.  We acknowledge that our study was limited to hospitalized patients, anddid not include |

|  | outpatient cases. This focus was driven by several factors:  First; Our primary objective was to evaluate NLR as a marker for assessing disease severity and predicting clinical deterioration and mortality among hospitalized COVID-19 patients. Hospitalized individuals represent a population with more severe disease presentations, where the prognostic value of NLR can be critically assessed and it is of importance.  Second; Was due to Data Availability and Reliability. The retrospective cohort study design utilized comprehensive data available from hospitalized patients at Kilimanjaro  Christian Medical Centre (KCMC). These data included detailed clinical records and laboratory results, which are often more complete and reliable for inpatients than for outpatients.  Thirdly; Given the scope and resources of our study, we focused on a well-defined and manageable cohort. The inclusion of outpatient cases would have required additional resources for data collection and management, which were beyond our capacity at the time. |
| --- | --- |

|  | We recognize the importance of evaluating NLR in a broader patient population, including outpatients. This would provide a more comprehensive understanding of NLR's utility across different clinical settings. Future studies should aim to include both hospitalized and outpatient cohorts to generalize the findings and further validate the prognostic value of NLR.  Despite the limitation, our study provides valuable insights into the prognostic role of NLR in hospitalized COVID-19 patients, which can inform clinical practice and help prioritize care for those at higher risk of adverse outcomes. |
| --- | --- |
| 4. Is NLR testing useful a few days after symptom onset? Do you suggest periodic NLR testing? | Thank you for your insightful questions. Our study provides initial insights into the timing and potential periodic testing of the  Neutrophil-to-Lymphocyte Ratio (NLR) in predicting mortality among COVID-19 patients.  Our study tested NLR levels at the time of admission, which typically occurs several days after symptom onset. The analysis showed a strong association between NLR levels at admission and in-hospital mortality.  As shown in Table 5, the duration since the first symptoms was significantly associated with in-hospital death. Patients admitted |

|  | earlier (within the first week of symptom onset) had a higher risk of mortality compared to those admitted later (beyond two weeks).  Based on our findings, we recommend considering periodic NLR testing during hospitalization. Monitoring NLR levels over time can provide ongoing insights into the patient's inflammatory status and help identify changes in their clinical condition.  However, a multicenter, large prospective study is needed to validate the periodic testing of NLR and to answer key questions regarding the frequency and optimal timing of NLR testing. |
| --- | --- |
| 5. The disparity in the population sizes between severe cases and milder cases in your study was notably low. Could this issue potentially impact the validity of the results? | Thank you for your observation. We acknowledge the disparity in population sizes between severe cases and milder cases in our study.  We acknowledge that the larger number of severe and critical cases could introduce a bias in the analysis, as these patients are more likely to have higher NLR values. This could potentially exaggerate the association between high NLR and adverse outcomes.  However; Despite the disparity, the significant P-values indicate a strong association between NLR levels and clinical |

|  | outcomes. Specifically, the P-value of <0.001 suggests that the observed differences in NLR distribution across clinical outcomes are not due to random chance.  Results from our study may be more applicable to similar hospital settings where severe and critical cases are more prevalent. However, caution should be exercised when generalizing these findings to outpatient or less severe populations, where the distribution of cases may be different.  Future research should aim to include a more balanced cohort to validate these findings across a broader spectrum of COVID-19 severity.  We appreciate your attention to this detail and hope this explanation addresses your concern. |
| --- | --- |
| 6. As age increases, various underlying conditions may arise in individuals, which could be significant contributing factors to mortality in COVID-19. | Thank you for highlighting this important point. We agree that age and the presence of underlying conditions are significant contributing factors to mortality in COVID19 patients. Our analysis took these factors into account by adjusting for them in our hazard ratio calculations. |
|  | In our study, we assessed the Neutrophil-toLymphocyte Ratio (NLR) and other factors associated with in-hospital death among patients admitted with COVID-19 at KCMC hospital.  Our findings show that even after adjusting for potential confounding factors, including age and underlying conditions, the NLR remained a significant predictor of in-hospital mortality. The adjusted hazard ratio (AHR) for NLR was 1.03 (95% CI 1.021-1.038, P < 0.001), indicating that higher NLR values are independently associated with increased risk of death.  This adjustment ensures that the association between NLR and mortality is not solely driven by age or comorbid conditions but reflects the prognostic value of NLR itself. Therefore, while age and underlying conditions are critical factors, NLR provides additional, independent prognostic information that can be useful in clinical decision-making.  We appreciate your attention to this detail and trust this explanation clarifies the clarity of our findings. |

| **Reviewer 2:** |  |
| --- | --- |
| 1. Kindly include any reference in introduction suggesting impact of NLR with clinical or supportive care during the hospital stay (use of steroids or any supportive therapy's that effect the NLR ratio) | Thank you for this feedback; The comment has been addressed on revised manuscript on page 5 line 115 to page 120. |
| 2. If there is any data on supportive care/therapy that impacted the NLR ratio in your results,? | Thank you for your question regarding the impact of supportive care or therapy on the NLR in our study results. It is important to clarify that all patients in our cohort received the same level of care as per the standardized treatment protocol at KCMC Hospital. This uniform approach included standard medical treatments and supportive care therapies recommended for COVID-19, adhering to both national guidelines and international best practices.  Due to the standardized treatment protocol implemented across all patient groups, there were no variations in the type of supportive care or therapies administered that could independently influence NLR values. Therefore, our analysis did not include a |

|  | comparison of NLR variations across different therapeutic interventions.  This standardization helps ensure that the observed variations in NLR were primarily reflective of the disease's natural progression and patient-specific responses rather than differences in treatment approaches. As such, any changes in NLR noted in our results are attributed to the clinical course of the patients rather than the effects of varying treatments. |
| --- | --- |
| 3. Provide/brief the different time points at which the baseline and follow-up tests were performed (are any standard criteria followed?) | Thank you for your inquiry regarding the timing and criteria for baseline and follow-up tests in our study. To clarify:  1. **Baseline Testing:** o **Timing:** All patients underwent a full blood count (FBC) at the emergency department upon admission. This was part of the initial assessment for all incoming COVID-19 patients.  o **Criteria:** The decision to perform this baseline FBC is aligned with our hospital’s standardized criteria for assessing the severity of illness in patients presenting with COVID-19 symptoms. This approach ensures that we |

|  | have a consistent set of data for all patients at the point of entry into the healthcare system.  2. **Follow-Up Testing:**  o **Timing and Criteria:** Our study did not consistently include follow-up tests in the analysis. There were significant variations in the timing and frequency of follow-up FBCs, mainly due to resource limitations and cost implications. Follow-up tests were not routinely performed unless there was a strong clinical indication. o **Reasons for Inconsistency:** A substantial number of patients did not receive follow-up tests. This inconsistency is primarily due to the resource constraints within our setting, where healthcare resources must be allocated judiciously. Consequently, follow-up blood tests are typically reserved for cases where clinical developments necessitate further evaluation. |
| --- | --- |
|  | Due to these factors, our analysis focused on the baseline FBC data to maintain consistency and reliability in our evaluation of NLR as a prognostic marker. This decision aligns with the practical and operational realities of managing a large volume of COVID-19 patients in a our resourceconstrained environment. |
| 4. Look into line number 232 (mentioned as xx) | Thank you for this feedback; The comment has been addressed on revised manuscript on page 10 line 259. |

| **Reviewer 3:** |  |
| --- | --- |
| **Reviewer #3:** Although the topic is no longer "hot", the article could be interesting for some readers. The study can be important because of NLR's simplicity and cost-effectiveness compared to other inflammatory markers. Is it possible to specify how they evaluated the covid waves (which time intervals were included for delimitation) in the conditions where there were statistically significant values between the waves?Given that the NLR is increased in many other conditions, in the absence of comorbidity evaluation, not only hypertension, is there no risk of bias?it would be interesting to evaluate the severity of the disease depending on the waves of COVID. In the first wave, all the patients were probably hospitalized, possibly also the asymptomatic ones or those with mild symptoms | Thank you for your insightful feedback. We appreciate your comments and the opportunity to improve our manuscript. Below are our detailed responses to your queries and suggestions:  **1. Clarification on COVID-19 Waves and Time Intervals:**  We appreciate your request for clarification on how COVID-19 waves were determined in our study. The waves were identified using the hospital's epicurve, which was generated based on the admission dates of patients with COVID-19.  **2. Risk of Bias Due to Lack of Comorbidity Evaluation:**  Your concern regarding the potential risk of bias due to incomplete comorbidity evaluation is well noted. In our analysis, we adjusted for several common comorbidities, including hypertension, diabetes, asthma/chronic lung disease, HIV, and other grouped comorbidities. However, other known comorbidities were present in smaller numbers and were not fully reviewed due to diagnostic and data limitations, which we acknowledge could contribute to residual confounding.  We have expanded the discussion to address this limitation, highlighting the impact that unmeasured or underrepresented conditions might have on NLR values and outcomes. This limitation underscores the need for careful interpretation of our results and suggests a need for further studies that can incorporate a broader evaluation of comorbidities in similar analyses.  **3. Severity of Disease Depending on COVID-19 Waves:**  You highlighted the importance of evaluating disease severity depending on the waves of COVID-19, noting that early waves likely included a broader spectrum of patients, including those with mild symptoms. We agree with this observation. Our analysis as seen in table 3 has incorporated analysis of severity of disease in relation to COVID-19 waves and it identified significant differences in disease severity across the waves. Specifically:   - Patients admitted during Wave 2 and Wave 4 had a significantly higher risk of severe disease compared to Wave 1, with adjusted relative risks (ARR) of 1.24 (95% CI: 1.04–1.47, p=0.015) for Wave 2 and 1.24 (95% CI: 1.02–1.50, p=0.029) for Wave 4. - In Wave 3, the increase in severity was observed but did not reach statistical significance after adjustment (ARR: 1.13; 95% CI: 0.93–1.36, p=0.216).   During the initial wave (Wave 1), hospitalization criteria were broader, often including asymptomatic and mild cases due to the early, cautious approach to managing the disease. In later waves, evolving knowledge and refined admission practices led to a higher proportion of moderate to severe cases being hospitalized, influencing the observed trends in disease severityWe hope these revisions address your concerns and improve the overall quality and clarity of our manuscript. Thank you once again for your valuable feedback. |

| **Reviewer 4:** |  |
| --- | --- |
| R**eviewer #4:** This study has been conducted to find the association of NLR with COVID-19 mortality and deterioration. Main issues of the manuscript are: 1. Association of NLR with COVID-19 mortality and a worse outcome is already well published. Many articles as pointed out by the authors and many more are available looking at the same findings giving the same conclusion. It is not clear what the current manuscript adds to the already known body of literature. Authors have failed to highlight any novelty of the findings or any additional information in the current manuscript.  2. Although laboratory data has been extracted from patient records, there is no reference to the findings or any association of outcome/NLR with these parameters. Only the comorbidities have been analysed.  Current data does not have novelty and it is not clear what additional information is provided to the scientific community. | Thank you for your insightful review and for highlighting key areas of concern regarding the novelty and contribution of our manuscript. We appreciate your feedback and have carefully addressed your comments as follows:   1. **Novelty and Contribution of the Current Manuscript:**   We understand your concern about the perceived lack of novelty in our findings, given that the association of NLR with COVID-19 mortality and adverse outcomes has been reported in numerous studies. However, it is important to emphasize that this study is among the few conducted in African settings, with only one other similar study available in the continent. The unique context of our study lies in its focus on a resource-constrained setting in Northern Tanzania, where access to advanced inflammatory markers and imaging tools is often limited.  This study is particularly relevant in local and similar settings where healthcare resources are constrained, and affordable, accessible, and simple prognostic tools like NLR are critically needed. Unlike studies conducted in high-income countries, our findings provide valuable insights into the utility of NLR in low-resource settings, where managing severe COVID-19 and other inflammatory conditions remains a significant challenge.  Furthermore, while COVID-19 might not currently be at its peak, the pandemic has demonstrated the importance of preparedness for recurring or emerging infectious diseases. NLR, as a simple, cost-effective marker, can play an essential role not just in current COVID-19 management but also in future pandemics and in monitoring other inflammatory conditions prevalent in our setting, such as bacterial infections, malaria, and other viral illnesses. We have highlighted this significance and the practical implications of our findings in the revised discussion section.   2. **Laboratory Data and Association with NLR/Outcomes:**   We acknowledge your point regarding the limited analysis of laboratory data beyond comorbidities. Although laboratory parameters were extracted from patient records, our primary focus was on evaluating the prognostic value of NLR due to its simplicity and accessibility compared to other laboratory markers that may not be routinely available in resource-limited settings.  Nevertheless, we have strengthened the discussion to include a broader context of how NLR correlates with other inflammatory markers as reported in the literature, even if direct associations were not specifically analyzed in our study due to the constraints of our data. This context further supports NLR’s practical utility in settings where more comprehensive laboratory testing may not be feasible. |
